# The mechanism and effect of repetitive transcranial magnetic stimulation for post-stroke pain

**DOI:** 10.3389/fnmol.2022.1091402

**Published:** 2023-01-06

**Authors:** Long-Jin Pan, Hui-Qi Zhu, Xin-An Zhang, Xue-Qiang Wang

**Affiliations:** ^1^College of Kinesiology, Shenyang Sport University, Shenyang, China; ^2^Department of Sport Rehabilitation, Shanghai University of Sport, Shanghai, China; ^3^Department of Rehabilitation Medicine, Shanghai Shangti Orthopaedic Hospital, Shanghai, China

**Keywords:** rTMS, stroke, pain, mechanisms, therapy, review

## Abstract

Post-stroke pain (PSP) is a common complication after stroke and affects patients' quality of life. Currently, drug therapy and non-invasive brain stimulation are common treatments for PSP. Given the poor efficacy of drug therapy and various side effects, non-invasive brain stimulation, such as repetitive transcranial magnetic stimulation (rTMS), has been accepted by many patients and attracted the attention of many researchers because of its non-invasive and painless nature. This article reviews the therapeutic effect of rTMS on PSP and discusses the possible mechanisms. In general, rTMS has a good therapeutic effect on PSP. Possible mechanisms of its analgesia include altering cortical excitability and synaptic plasticity, modulating the release of related neurotransmitters, and affecting the structural and functional connectivity of brain regions involved in pain processing and modulation. At present, studies on the mechanism of rTMS in the treatment of PSP are lacking, so we hope this review can provide a theoretical basis for future mechanism studies.

## 1. Introduction

Stroke is a disease with high morbidity, disability, and mortality worldwide (Lou et al., 2020). The many sequelae after stroke include motor dysfunction, pain, cognitive dysfunction, and paresthesia. Pain is one of the common sequelae after stroke and affects the quality of life. The incidence of post-stroke pain (PSP) is reported to be 10–45.8% (Yang and Chang, 2021; Zhang et al., 2021). PSP can manifest in many forms, including central PSP (CPSP), painful spasms, hemiplegia, tension headaches, and musculoskeletal pain (Delpont et al., 2018; Torres-Parada et al., 2020; Yang and Chang, 2021). CPSP is a neuropathic pain syndrome with challenging treatment due to vascular lesions of the somatosensory pathways in the brain (Boivie et al., 1989; Jang et al., 2019). The reported incidence of CPSP is 1–12% (Hansen et al., 2012). In most CPSP patients with dysesthesia, hyperalgesia, and paresthesia (Klit et al., 2011), pain can be characterized by spontaneous continuous pain (usually burning pain, squeezing, penetrating, and numbness) or spontaneous intermittent pain (Kumar et al., 2009). Pain severity is variable and involves temperature changes and emotional stress; movement aggravating pain, rest, and distraction can reduce pain (de Oliveira et al., 2012). CPSP has negative effects on mood, sleep, rehabilitation, and quality of life in stroke patients. PSP is often managed with a combination of medications, cognitive behavioral therapy, psychotherapy, and/or other non-pharmacological treatments (repetitive transcranial magnetic stimulation [rTMS] (Chen et al., 2022), electromotor core cortical stimulation [EMCS], and deep brain stimulation [DBS] (Cruccu et al., 2007; Elias et al., 2018)).

The management of CPSP remains challenging, and some evidence-based studies suggest limited pain relief even with high doses of different medications (Scuteri et al., 2020; Choi et al., 2021). Invasive and non-invasive neurostimulation can provide at least moderate chronic pain relief. However, invasive treatment entails risks, and non-invasive transcranial magnetic stimulation is the current treatment option for many patients (Hosomi et al., 2015; Yang et al., 2022).

rTMS provides a non-invasive and non-painful means of central nervous modulation for studying and treating neuropathic pain states. The physical principle of rTMS is electromagnetic induction. Current pulses pass through induction coils on the scalp to generate magnetic pulses and transmit them to the brain. Magnetic pulses delivered by the coil induce an electric field in the cortex, which activates neurons in the cerebral cortex (Ridding and Rothwell, 2007; Afuwape et al., 2021). Transcranial magnetic stimulation activates remote, interconnected parts of the brain in addition to targeted areas (Hallett et al., 2017).

Few reviews have summarized the effect and mechanism of rTMS on PSP. This work reviewed recent studies on rTMS improving PSP and analyzed the analgesic effect and potential mechanism of rTMS on PSP. This review discusses the therapeutic effects of different treatment parameters, hoping to provide help for the formulation of standard rTMS treatment of PSP. It also discusses the possible mechanism of rTMS in the treatment of PSP. We hope to provide more references for future research on the mechanism of rTMS.

## 2. Effect of rTMS on PSP

Pain is one of the common sequelae after stroke, and pain usually occurs on the opposite side of the central lesion, mainly in the upper extremities. Burning, hurting, tingling, freezing, crushing, shooting, or stabbing sensations are common descriptors. It is often affected by factors such as temperature, psychological stress, fatigue, and physical exercise. In clinical practice, CPSP can be difficult to distinguish from other types of PSP, such as hemiplegic shoulder pain, painful cramps, tension headaches, and other musculoskeletal pain (Klit et al., 2009). CPSP can impair quality of life, disrupt recovery, interfere with sleep, affect mood (produce depression or anxiety), and occasionally lead to suicide. The treatment of refractory CPSP usually adopts comprehensive treatment, but the treatment effect is often not ideal. Non-invasive treatment has received extensive attention in recent years in improving refractory CPSP. rTMS is a safe, non-invasive, tolerable, and effective mode of therapeutic intervention. It delivers many pulses continuously at a constant rate and is widely used clinically (Hosomi et al., 2015; Gu and Chang, 2017). More studies on the effect of rTMS on CPSP are detailed in Table 1.

**Table 1:** Major findings of repetitive transcranial magnetic stimulation in post-stroke pain studies.

**Author, year**	**Country**	**Study type**	**Stroke type**	**PSP type**	**Sample (size, sex, age)**	**rTMS site**	**Frequency/Intensity**	**Duration**	**Pulses**	**Intertrain interval**	**Analgesic effect**
de Oliveira et al. (2014)	Brazil	RCT	ALL	CPSP	21,10 M,11 F Real 55 ± 9.67 Sham 57.8 ± 11.86	PMC/DLPFC	10 HZ/120% of RMT	10 sessions	1,250	25 s	No effect pain relief by VAS
Hosomi et al. (2013)	Japan	Cross-over	ALL	CPSP	21, 12 M, 9 F 59.6 ± 9.0	M1	5hz/90% of RMT	10 sessions	500	50 s	Pain relief by VAS
Malfitano et al. (2021)	Italy	Case series	Ischemic stroke	CPSP	1, 1 F, 32	M1	10 Hz/90% of RMT	10 sessions	2,000	5 s	Pain relief by NRS
Zhao et al. (2021)	China	RCT	ALL	CPSP	38, 21 M, 7 F, Real: 50.1 ± 11.34, Sham: 48.9 ± 11.51	M1	10 Hz/80% of RMT	18 days	1,500	3 s	Pain relief by NRS and MPQ
Choi-Kwon et al. (2017)	Korea	RCT	ALL	HSP	24, 13 M, 11 F 59.0 ± 8.0	M1	10 Hz/90% of RMT	10 sessions	1,000	55 s	Pain relief by NRS
Kobayashi et al. (2015)	Japan	Cross-over	ALL	Pain in the paretic extremities	18, 12M, 6F, 63.0 ± 9.9	M1	5 Hz/90%RMT	12 weeks	500	50 s	Pain relief by VAS
Hasan et al. (2014)	UK	Cross-over	ALL	CPSP	14, 10 M, 4 F 57median	M1	10 Hz/80%− 90%RMT	5 sessions	2,000	60 s	Pain relief by NRS
Ohn et al. (2012)	Republic of Korea	Case series	ALL	CPSP	22, 13 M, 9 F 54.9 ± 9.0	M1	10 Hz/90% of RMT	5 sessions	1,000	55 s	Pain relief by VAS
Ojala et al. (2021)	Finland	RCT	ALL	CPSP	17	M1/S2	10 Hz/90% of RMT	10 sessions	5,050	50	Pain relief by NRS

CPSP, central PSP; PSP, Post-stroke pain; HSP, hemiplegic shoulder pain; M, male; F, female; RMT, resting motor threshold; M1, primary motor cortex; DLPFC, dorsolateral prefrontal cortex; PMC, premotor cortex; VAS, visual analog scale; NRS, numerical rating scale; S2, secondary somatosensory cortex.

### 2.1. Analgesic effect of rTMS

#### 2.1.1. HF-rTMS has good analgesic effect

At present, there is no unified standard for the treatment parameters of rTMS in the treatment of PSP. Different treatment parameters (stimulation frequency, stimulation site, and treatment duration) have varying analgesic effects. Traditionally, low-frequency (LF) rTMS (defined as stimulation at frequencies below 1 Hz) has been shown to reduce cortical excitability, whereas high-frequency (HF) rTMS (stimulation at frequencies over 1 Hz) has the opposite effect (Wassermann, 1998; Wagner et al., 2007; Bai et al., 2022). Multiple studies have shown that HF-rTMS provides better pain relief than LF stimulation (Cruccu et al., 2007; Borckardt et al., 2011; Pazzaglia et al., 2018). In previous studies on the analgesic effect of rTMS on PSP, HF-rTMS (5–20 Hz) was found to be effective in relieving pain in PSP. Multi-session and longer interventions can produce better analgesic effects than single sessions and short interventions (Ohn et al., 2012; Hosomi et al., 2013; Ramger et al., 2019).

#### 2.1.2. Significant analgesic effect by targeting the M1 site

There are also significant differences in the analgesic effects of different stimulation targets. At present, the stimulation target selected in most studies is the M1 site, and the analgesic effect in this site is more intuitive and significant than that in other sites. Hirayama et al. (2006) applied 90% of RMT rTMS to the premotor cortex (M1), the primary somatosensory cortex (S1), the premotor area (preM), and supplementary motor area. Ten trains of 10 s 5 Hz TMS pulses were applied to each site, with 50's intervals between each train. Visual analog scale and the McGill Pain Questionnaire (SF-MPQ) scores were employed to determine the effect of RMT rTMS on pain, the results showed that stimulation at the M1 site has a more significant analgesic effect than that at other sites. In addition, de Oliveira et al. (2014) selected the premotor cortex/dorsolateral prefrontal cortex (PMC/DLPFC) for stimulation, each stimulation intensity was 120% of RMT, 1,250 pulses per session, stimulation interval 25 s, a total of 10 sessions, VAS score was measured after each session, and the results showed no significant pain relief effect in CPSP. Thus, this result may be related to its small sample size, but the negligible analgesic effect shows that PMC/DLPFC is not a good choice for the treatment of CPSP. However, in a recent randomized controlled trial, some scholars compared the analgesic effect of S2 and M1 on CPSP, after 10 times of stimulation at 10 HZ, each with an intensity of 5,050 pulses, the numerical rating scale (NRS) was used to measure the degree of pain of the patient, the results showed that both targeted S1 and M1 stimulation had short-term analgesic effects, but there was no difference compared to the sham group, suggesting a strong placebo effect (Ojala et al., 2021).

#### 2.1.3. rTMS has analgesic effects on various types of pain

In addition to common neuropathic pain after stroke, HF-rTMS (10 Hz) also has a significant relieving effect on post-stroke shoulder pain, some scholars have applied rTMS with 90% of RMT and pulse 1000 to M1 for 10 consecutive sessions of treatment, the NRS score was measured on the first day, the first week, the second week, and the fourth week. The results showed that rTMS can significantly relieve HSP and maintained it for about 4 weeks, indicating that rTMS also has a certain relieving effect on peripheral pain (Choi and Chang, 2018). Studies on rTMS are mainly aimed at chronic CPSP, which may be caused by cognitive impairment in early-stage patients, leading to difficulty in diagnosis or first-line treatment of CPSP. According to expert consensus, HF-rTMS (>5 Hz) can provides moderate pain relief in chronic CPSP (Leung et al., 2020). But rTMS studies on acute or subacute CPSP are few. In a case study, 10 Hz rTMS was applied to the M1 site of a subacute CPSP patient for 2,000 pulses each time, with an intensity of 90% of RMT, for a total of 10 sessions, and the pain levels were measured before, after stimulation, and 1 month after stimulation. The results showed that the level of post-stimulation was significantly lower than the baseline level, suggesting that rTMS had the same analgesic effect on acute CPSP (Malfitano et al., 2021).

The analgesic effect of rTMS on PSP is positive correlates with frequency and treatment duration, and the M1 site as a stimulation target has a better therapeutic effect than other sites. rTMS has a good analgesic effect on pain, regardless of whether the pain is neuropathic, peripheral, acute or chronic.

### 2.2. rTMS has antidepressant effects

PSP is often accompanied by depression and anxiety, which aggravates the pain and hinders the recovery process. In many studies, pain relief was accompanied by depression improvement, suggesting that depression, and anxiety may influence the analgesic effect of rTMS. Ohn et al. (2012) applied 10 Hz rTMS (M1) to 22 patients with CPSP, and gave rTMS with an intensity of 90% of RMT and 1000 pulses. After 5 days of continuous treatment, the responders' VAS score and HDRS score decreased significantly. Results showed the lower the HDRS score at baseline, the more significant the analgesic effect, indicating a relationship between the improvement of depressive mood and pain relief. Galhardoni et al. (2019) applied 10 Hz rTMS to the anterior cingulate cortex (Hasan et al., 2014) and posterior superior insula (PSI), a total of 16 sessions lasted for 12 weeks, the stimulation intensity was 90 of RMT, 1,500 pulses, and the results showed that both ACC stimulation and PSI stimulation produced pain relief but no significant difference. In contrast, ACC stimulation had a more significant anxiolytic effect than PSI simulation. However, little research has been conducted in this area, and more studies are needed in the future to demonstrate the relationship between mood improvement and pain relief.

## 3. Mechanism of rTMS for PSP

### 3.1. rTMS changes cerebral cortical excitability

**Figure 1:**
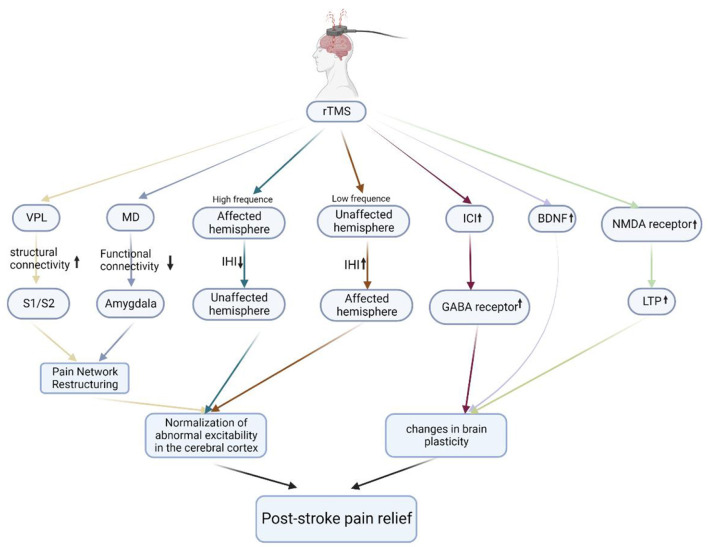
The mechanisms of rTMS in the treatment of PSP. Schematic illustration of the underlying mechanism of rTMS in the treatment of PSP. rTMS modulates the abnormal excitability of the cerebral cortex by modulating the pain network, improving interhemispheric inhibition, and increasing the number of GABA receptors, BDNF expression, and the number of NMDA receptors to alter brain plasticity, ultimately relieving PSP. VPL, ventral posterolateral nucleus; S1, primary somatosensory cortex; S2, secondary somatosensory cortex; MD, mediodorsal nucleus; IHI, interhemispheric inhibition; ICI, intracortical inhibition; GABA receptor, gamma-aminobutyric acid receptor; BDNF, brain-derived neurotrophic factor; NMDA receptor, N-methyl-D-aspartate receptor; LTP, long-term potentiation.

Although rTMS has been reported to have a significant analgesic effect on PSP, the underlying mechanism of its therapeutic effect has not been fully defined. The rTMS-promoting recovery of cortical excitability has been reported by many studies. Spontaneous pain in CPSP may be related to hyperexcitability or spontaneous firing of missing neurons in the thalamus or cortex (Vestergaard et al., 1995; Walton and Llinás, 2010), and pain relief after rTMS treatment of stroke is often accompanied by recovery of abnormal cortical excitability. This is considered one of the possible mechanisms for rTMS to treat PSP (Hosomi et al., 2013). It may involve the functional reorganization of various parts of the cerebral cortex, the recovery of abnormal inhibition between the cerebral hemispheres, and the transmission of some neurotransmitters. The mechanism of rTMS in the treatment of PSP is shown in Figure 1.

#### 3.1.1. rTMS affects functional reorganization of the brain

CPSP typically occurs weeks or months after stroke. This late-onset feature suggests that the mechanism of CPSP development is gradual, possibly through maladaptive pain network reorganization or plastic changes (Hosomi et al., 2015). CPSP is often associated with strokes that occur around somatosensory pathways, including the ventral posterolateral nucleus (VPL) of the thalamus, anterior occipital nucleus, and lateral medulla. CPSP may also involve changes in medial emotional pathways, including the amygdala, anterior cingulate cortex, and insular cortex (Sprenger et al., 2012; Vartiainen et al., 2016). CPSP is thought to be caused by maladaptive reorganization between different regions of the brain, which neuromodulation techniques can normalize and treat (Chen et al., 2022). Evidence from animal studies suggests that CPSP reduces functional connectivity between the VPL and S1 (primary somatosensory cortex)/S2 (secondary somatosensory cortex) (responsible for perceiving pain location, intensity, and duration) and increases functional connectivity (responsible for the attentional, cognitive, and emotional aspects of pain evaluation) between the mediodorsal nucleus (Tarragó et al., 2016) and the amygdala. rTMS therapy relieves this abnormal connection (Kadono et al., 2021), so we speculate that this may be one of the mechanisms of rTMS pain relief.

#### 3.1.2. rTMS affects interhemispheric inhibition

The mechanism of CPSP is unclear, but theories of central disinhibition have been proposed (Head and Holmes, 1911). Damage to the lateral thalamus is hypothesized to free the medial thalamus from control, inducing spontaneous or atopic pain. This is similar to the later proposed model of interhemispheric inhibition (IHI) (Duque et al., 2005), which assumes balanced inhibition between the hemispheres of a healthy brain. Although IHI theory is more commonly used to explain the recovery of dyskinesia after stroke, some scholars believe that IHI may also be involved in the CPSP mechanism (Morishita and Inoue, 2016). On the basis of IHI theory and the fact that M1 transcranial magnetic stimulation inhibits CPSP, we speculate that inhibitory signals from the contralateral hemisphere may suppress the activity of M1 in the ipsilateral hemisphere, and the mechanism of pain suppression in patients with CPSP may malfunction. The presence of post-stroke lesions results in reduced M1 excitability in the affected hemisphere, thereby reducing its neural output, including IHI to M1 in the unaffected hemisphere. This resulted in a relative increase in the excitability of M1 in the contralateral hemisphere and increased neural output, thereby increasing the IHI from M1 in the contralateral hemisphere to M1 in the affected hemisphere and inhibiting the excitability of M1 in the affected hemisphere (Gerges et al., 2022). A recent study found that rTMS induces an Increase in IHI in the affected hemisphere to the contralateral hemisphere, thereby relieving pain (Alhassani et al., 2019). Therefore, LF-rTMS of the unaffected hemisphere may reduce inhibition to the affected hemisphere. In contrast, HF-rTMS of the affected hemisphere increases inhibition to the unaffected hemisphere, normalizing the excitability of the cerebral cortex and finally achieving the effect of pain relief. Discussions of our theory focus more on movement disorders after stroke, and further research and discussion are needed on PSP.

#### 3.1.3. RTMS affects GABAergic neuron transmission

Various experimental studies have highlighted the reduction of GABAergic neurotransmission in the central nervous system as the main reason for chronic neuropathic pain (Neto et al., 2006; Yang et al., 2019). In animal models of neuropathic pain, decreased GABAergic tone was found at the level of the dorsal spinal cord, thalamic sensory nuclei, and somatosensory cortex, which resulted in neuronal hyperactivity in the sensorimotor cortex (Guilbaud et al., 1992; Paz et al., 2010). Intracortical inhibition (ICI) is thought to reflect the function of interneurons within M1; ICI and intracortical facilitation (ICF) may reflect GABAergic inhibitory interneurons, especially GABA function (Ziemann et al., 1996; Reis et al., 2008; Lanza et al., 2020). Previous studies have shown that HF-rTMS can increase ICI and ICF, and this change is associated with PSP relief (Lefaucheur et al., 2006; Hosomi et al., 2013). HF-rTMS has been observed to enhance GABAergic synaptic connections (Lefaucheur et al., 2006); therefore, rTMS can relieve PSP through this mechanism of enhancing GABAergic neuron transmission.

### 3.2. rTMS induces changes in brain plasticity

Chronic pain states are known to be associated with neuroplastic changes, and the development of neuropathic pain states may involve changes in supraspinal canal function associated with pain perception (Lorenz and Casey, 2005; Maihöfner et al., 2006; Thompson and Neugebauer, 2019). The neurotrophic factor BDNF is closely related to neuronal plasticity, which is important for neuropathic pain recovery. Increased expression of BDNF has been observed in subacute and chronic stroke patients with neuropathic pain, suggesting that BDNF is involved in pain recovery after stroke (Siotto et al., 2017). The effect of rTMS on brain plasticity is mainly through two forms of long-term potentiation (LTP) and long-term depression (LTD) (Hoogendam et al., 2010). LTP can durably enhance synaptic strength from days to months, whereas LTD causes a secular decrease in synaptic strength (Duffau, 2006). The induction of LTP and LTD may be related to NMDA receptors, NMDA receptors contain ion channels that are blocked by resting magnesium ions, but membrane depolarization unblocks this channel, allowing calcium ions to enter the postsynaptic neuron and finally induce LTP (Cooke and Bliss, 2006). NMDA receptor activation is also involved in LTD, but in a different way. Rapid increases in postsynaptic calcium content induce LTP, whereas small and slow flows of calcium induce LTD. Previous studies have also demonstrated that rTMS can increase the number of NMDA receptors in the ventromedial thalamus, amygdala, and parietal cortex (Lisanby and Belmaker, 2000). Accordingly, we speculated that rTMS modifies the plasticity changes of the nervous system by increasing the expression of NMDA receptors. BDNF is a “classical” neurotrophic factor that has been shown to be closely related to neuronal plasticity and neuropathic pain. Many studies have shown that serum BDNF is inversely correlated with pain levels (Zhao et al., 2021). In a previous study (Dall'agnol et al., 2014), patients with CPSP experienced significant increases in serum BDNF levels and less pain after 3 weeks of rTMS treatment. In another experiment (Zhao et al., 2021), 10 HZ rTMS was applied to 40 patients with acute CPSP. After 3 weeks of treatment, the pain was significantly relieved and accompanied by an increase in serum BDNF. This result was consistent with previous findings, suggesting that the pain relief effect of rTMS may be related to an increase in BDNF.

## 4. Conclusion

rTMS is the preferred non-invasive, non-drug therapy for PSP, which is safer than invasive therapy and more easily accepted by patients. rTMS has good analgesic effects on both neuropathic and peripheral pain after stroke, which may be related to the recovery of cortical excitability, changes in brain plasticity, and pain-related mood and sensory improvement. Most of the current research results show that HF, M1, and multiple courses of rTMS have better therapeutic effects than LF and a single session of rTMS. However, there is currently no standard treatment plan for rTMS treatment of PSP. The mechanism by which rTMS relieves pain has yet to be determined. This review discusses the possible mechanism of rTMS to improve pain, and it is hoped to provide a theoretical basis for future research.

## Author contributions

X-QW and X-AZ: draft conception, project administration, and funding acquisition. L-JP, H-QZ, X-QW, and X-AZ: writing, reviewing, and editing. All authors contributed to the article and approved the submitted version.
